# The Multivariate Regression Models Suggested as Standardising Tools for Categorising Solitarious and Gregarious Groups of the Main Pest Locust, *Schistocerca gregaria*, Produce Reproducible Results

**DOI:** 10.3390/insects15020102

**Published:** 2024-02-01

**Authors:** Somia Saadi, Noureddine Bakkali, Rubén Martín-Blázquez, Abdelmounim Badih, Mohammed Bakkali

**Affiliations:** 1 Departamento de Genética, Facultad de Ciencias, Universidad de Granada, Fuentenueva S/N, 18071 Granada, Spain; biosoumia23@gmail.com (S.S.); noureddine.bakkali.2015@gmail.com (N.B.); 2 Estación Biológica de Doñana, Consejo Superior de Investigaciones Científicas (CSIC), Avenida Américo Vespucio 26, Isla de la Cartuja, 41092 Sevilla, Spain; rmartinblazquez@gmail.com; 3 Departamento de Ciencias Naturales, Agora Granada College International School, Urbanización Llanos de Silva S/N, 18230 Atarfe, Spain; abdelmounim.badih@granadacollege.es

**Keywords:** locust, phase change, outbreak, solitarious, gregarious, model

## Abstract

**Simple Summary:**

Locusts can be at a state called the solitarious phase, associated with harmless populations, or at the gregarious phase, associated with outbreaks. Given the importance of the phenomenon, researchers are trying to reveal its molecular basis and find ways to tackle it. For this, assessing the phase state and comparing locusts is essential, and researchers have thus far used different formulae. To address the problem presented by the lack of standardised tools for such an essential task, we previously suggested two models as tools for standardising the method for assessing the main pest locust, *Schistocerca gregaria*. However, a theoretical work later cast doubts on the validity of such models and predicted that they would not work as well on future samples as in their initial application. Here, we use additional, different *S. gregaria* samples to test and assess the performance of these models. The results reaffirm the validity of the results of our previous work, since the models performed just as well on the present samples as they did in the previous ones. The models are thus reinstated as potential tools for standardising the way solitarious and gregarious *S. gregaria* samples are assessed and compared.

**Abstract:**

Outbreaks of the desert locust *Schistocerca gregaria* affect some of the poorest parts of Africa, with devastating outcomes. The key to understanding and dealing with this problematic adaptation to environmental changes is comparing gregarious and solitarious locusts, either in nature or in laboratories. Categorising locusts and detecting changes in their phase status is key to such comparisons, which have been hitherto based on applying mathematical models that use behavioural parameters and that each laboratory has to build anew for each experiment. All the models used thus far are different from one another. This implies differences in the tools used for the different experiments and by the different laboratories and, thus, potential noise in the results and interpretations. Standardising the way locusts are categorised is necessary if we want to reduce noise and errors. It is crucial if we seek to make the results and interpretations transferable and comparable between experiments and laboratories for such an important research area. To tackle this problem, we suggested two models as possible standardising tools. However, the problem of a lack of standardised tools re-emerged due to the doubts cast on the validity of those models. Here, we use samples from independent *S. gregaria* populations in order to test and validate those models. We discuss how successful the two models were at categorising solitarious, intermediate (transient), and gregarious nymph and adult *S. gregaria* samples. We highlight shortcomings and make more specific recommendations on the use of those models based on the precision differences they show when categorising solitarious and gregarious *S. gregaria* nymph and adult samples. Overall, both models have proven to be valid since their results were largely replicated and seem reproducible.

## 1. Introduction

Locust outbreaks recurrently devastate vast regions around the globe. Different species are responsible for such devastation, including *Locusta migratoria* (mainly in Asia) [1], *Melanoplus sanguinipes* (in North America) [2], *Schistocerca piceifrons* and *Schistocerca cancellata* (in Latin America) [3,4], *Dociostaurus maroccanus* (in Southern Europe and Central Asia) [5] and *Chortoicetes terminifera* (in Australia) [6], among other species. *Schistocerca gregaria*, the desert locust, is considered the most devastating pest locust due to its broad distribution range (from Northwest Africa to India) [7,8] and to the damage it causes to regions that are already affected by extreme climates, poverty, war, and corruption [9]. The especially pressing need to find ways to prevent, protect, and help those areas against locust outbreaks makes *S. gregaria* a species of interest to science.

Like other locust species, *S. gregaria* is usually found living in low-population-density conditions, where individuals are at a state referred to as the solitarious phase, at which locusts display sedentary behaviour, social avoidance, and usually inconspicuous coloration. When climatic conditions change and become favourable, locusts experience an increase in their population size, and a subsequent series of physiological (e.g., [10,11,12,13]), behavioural (e.g., [14,15,16,17]), and morphological changes (e.g., [18,19]) occur. Their mobility increases, and they start aggregating onto the dense bands and massive swarms associated with their being in the gregarious phase during outbreaks. The differences between the two states (phases) of the locusts are adaptive responses to the change in the living environment that the locusts suffer. They co-occur in long-term solitarious and gregarious individuals. However, these differences are decoupled in short-term gregarious and solitarious locusts, meaning that solitarious locusts that have been just gregarised and gregarious locusts that are just solitarised will change behaviour but will not change colour and morphometry until their next moult(s) (e.g., [14,15,16,17,20]).

Molecular and genetic testing is becoming a common strategy in all the fields of research that study *S. gregaria* (e.g., [21,22,23]), and we are entering into an era of functional testing in non-model species. Accordingly, research on *S. gregaria* is, as predicted in [24], increasingly shifting towards functional studies in a quest for a better understanding and tackling of locust plagues. Comparing solitarious and gregarious locusts and assessing changes in the phase status of the locusts is key to such research. Logistic regression models are usually used for such assessment and comparisons of the locust phase status. However, new models have to be built by each laboratory and for each piece of experiment, and no agreed-upon model (tool) is available for standardising the way that the locust phase is assessed and how solitarious and gregarious locusts are categorised (e.g., [16,25,26]). In light of this serious problem, in 2017, we measured several morphological and behavioural traits (variables) from solitarious and gregarious *S. gregaria* locusts, and we assessed the correlation between the changes in each of these variables and the locust phase. The variables that correlated the most with the locust phase were used for model building, based on logistic regression formulae. The results of those formulae were transformed into probabilities of a locust being gregarious, where P_greg_ = 0 was assigned to solitarious and P_greg_ = 1 was assigned to gregarious locusts.

After testing on additional solitarious and gregarious locusts, we suggested the use of two logistical regression models as standardised tools to detect changes in *S. gregaria* locust phase status or to distinguish between solitarious and gregarious *S. gregaria* samples [27].

One of those two models, the *Sg_extended_corrected* model, is based not only on behavioural variables but also on selected morphological variables that were proven to correlate with the long-term phase status of *S. grearia* locusts. The other model, the *Sg_non-morphometric* model, included selected behavioural variables only. The first model was intended for experiments that compare different locust groups, including long-term solitarious and long-term gregarious locust groups and groups of nymphs that moulted during the experiment (i.e., animals whose morphology is not the same between the two ends of the comparison). The second model was intended for use on animals that do not change morphology between the two ends of the comparison (i.e., just solitarised and just gregarised locust groups that do not moult or change morphology during the experiment). A novelty that we introduced, compared to previous models, was that we normalised the movement-related variables—which are function of body size—using the animal’s femur (leg) size. The models were optimised and tested on different locust cages of different states (raised at different densities), sexes, developmental stages (adults and nymphs), and origins (populations). They were reasonably able to distinguish solitarious from gregarious *S. gregaria* samples in all the different sets. Nonetheless, the performance of the *Sg_non-morphometry* model was better than that of the *Sg_extended_corrected* model, with the latter deemed useless for adult samples. We therefore wrote that “we suggest using the ‘*Sg_extended_corrected*’ model (that includes morphometric variables) for comparing different S. gregaria nymph samples. For testing adults or the same nymphs at different time points (if they do not molt), we suggest using the *‘Sg_non-morphometric’* model (that does not include morphometric variables)” [27].

In silico re-application of the models to our data successfully reproduced the model’s outcomes, at least “with two-digit accuracy or better” [28]. Still, interpretations in the same work [28] cast doubts on the standardization possibility and on how the models were built, and led to predicting that the model “will not predict future observations as well as it appeared to predict on the present sample” [28]. Hence, the problem of the lack of valid tools to standardise how we compare and assess the phase status of *S. gregaria* locust samples re-emerged.

Since the prediction in [28] was based on theoretical analyses and interpretations and not on testing real locusts, here, we take advantage of the re-population and renewal of our laboratory locust colony—which was annihilated during the COVID-19 restriction period—in order to directly test and answer the question of whether the models would work in additional *S. gregaria* samples that are different from the ones tested in [27]. In the affirmative case, the models would be re-established as valid and possible standardising tools for the research community to use. Otherwise, the search for new tools would still be needed.

This work shows that, contrary to the prediction in [28], when used on additional, different, samples of actual solitarious and gregarious *S. gregaria* locusts, the models worked with similar performance and limitations as they did in [27]. We discuss the reproducibility of the results and validity of the models, and we refine our recommendations for using these models. We also signal the model’s advantages and limitations for time savings and, more importantly, for standardising the very important task of categorising *S. gregaria* samples for relevant research on that locust’s phase change and population outbreaks.

## 2. Materials and Methods

We used *S. gregaria* of different phases (solitarious, transient, and gregarious), stages (nymphs of different stages and adults), and sexes. The locusts were unrelated to the 12 sets of two origins that we used for the building and initial testing of the models in [27]. The personnel that reared the locusts and measured the variables and video-recorded the locusts was different from those who conducted the work in [27]. Furthermore, the personnel that applied the models using the gathered morphometric and behavioural data were unaware of (blind to) the locust rearing conditions and phases.

We raised the locusts at different densities, as described in [27]. Briefly, all the locusts shared some rearing conditions (31 degrees Celsius temperature, 14:10 light/dark period, and the same food—cabbage and corn flakes), while they differed in other rearing conditions (the phase-related ones). The solitarious locusts were raised each in an individual small cage in complete visual, mechanical (contact), and chemical (olfactory) isolation from other locusts, in order to prevent them from turning gregarious. The gregarious locusts were raised in groups in large cages that allow mechanical, visual, and olfactory contact between the locusts. The transient locusts were from a gregarious colony, but they were reared for one generation at lower density. The videos were taken at midday in the same room and a 60 × 60 × 60 cm observation arena as described in [27]. Two opposite sides of the observation arena were respectively separated from the control and stimulus using transparent glass. The control was an empty 60 × 60 × 60 cm wooden rearing cage that had a 60-watt light bulb turned on in the upper part of its back wall. We did not place locusts in that cage, in order to avoid accumulation of pheromones and other chemicals that might attract locusts. The stimulus was another 60 × 60 × 60 cm cage containing tens of gregarious locusts and a 60-watt light bulb turned on in the same place as in the empty (control) cage at the opposite side of the observation arena. The behavioural data were extracted from the videos using the R script provided in [27]. The morphometric data were measured for each individual after (not before) its video-recording—in order to avoid disturbing its behaviour. The probability of a locust being gregarious was separately calculated for each individual using the *Sg_extanded_corrected* and the *Sg_non-morphometric* models from [27]. The animals were tested only once—in order to avoid the effect of habituation. Animals that jumped straight to the arena’s walls were discarded; otherwise, the data were considered no matter how the animal behaved. The personnel that applied the models were blind to the state of the locusts, and no post-analysis filtering was applied to the data (i.e., data were considered even when they did not fit the individual locust’s phase status), in order to avoid subjective manipulation or bias of the results. Nymphs and adults were uncontrolled mixes of different sexes—since the models do not distinguish between sexes—and the nymphs were uncontrolled mixes of different stages—since the models do not distinguish between nymphal stages. First and second instar nymphs were not used in order to avoid the possible effect of mechanical damage when handling them; they are too small and fragile for the handling required for the morphometrical measurements and behavioural observations needed for the present work.

In total, we tested 279 additional *S. gregaria* locusts grouped into 11 sets: two sets of solitarious nymphs, three sets of solitarious adults, four sets of gregarious nymphs, and two sets of gregarious adults. In addition, we also tested two sets of gregarious adults and nymphs that were transferred since eclosion to lower density cages—referred to as transient adults and transient nymphs, respectively.

All the variables were already analysed as to their association with the locust phase, and they, as well as the methods used here, were as specified in [27]. As a reminder, the variables and formulae of both models are in Table 1 and, as the results of both models are interpretable at the group/sample not the individual level, the models were assessed based on the mean values they assigned to each locust set (i.e., the means obtained for solitarious nymphs, transient nymphs, gregarious nymphs, solitarious adults, transient adults, and gregarious adults).

In order to assess the differences between the locust samples, and before applying the models, the morphometric and behavioural variables that we extracted from the locust samples were analysed. For that, we used the ANOVA design: variable ~ Phase + Age + Sex + Phase * Age, where phase had the levels gregarious, solitarious, and transient; age had the levels adult and nymph; and sex had the levels female and male. The sex ratio of the samples was tested using the Chi squared test, and the Mann–Whitney test was used in Statistica 8.0 to compare the outcomes of each model between sample types. All other statistical analyses were performed using R project v4.1.2.

## 3. Results

### 3.1. Sets of Locusts Used Are Significantly Different from Each Other

**Figure 1:**
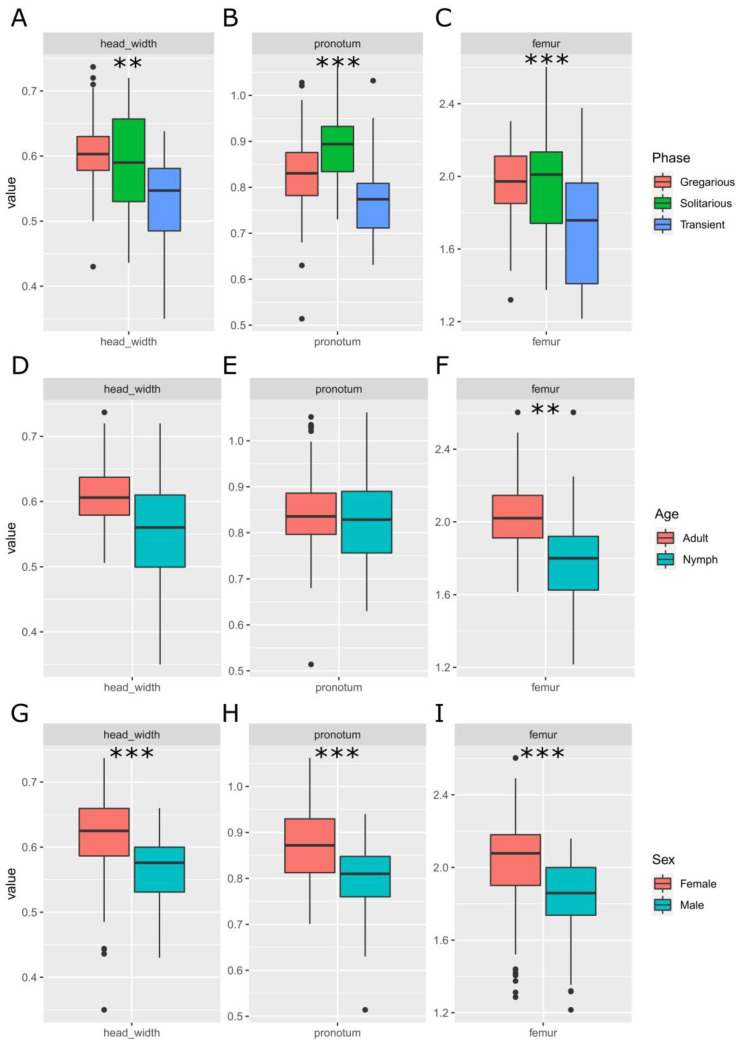
Effect of the phase (**A**–**C**), age (**D**–**F**), and sex (**G**–**I**) on the morphometric traits (head width (**A**,**D**,**G**), pronotum (**B**,**E**,**H**) and femur length (**C**,**F**,**I**)) used for building the models for inferring the phase of groups and populations of the desert locust *Schistocerca gregaria*. The *X* axis is the locust type (sex, age, or phase), and the *Y* axis is the size of the trait in mm. The solitarious, transient, and gregarious adult and nymph samples are different samples, not the same sample in different states. **: *p* < 0.01, ***: *p* < 0.001.

Measurements of head width, pronotum length, and femur length showed statistically significant differences between phases (Figure 1A–C) and sexes (Figure 1G–I). Solitarious females showed higher values for femur and pronotum length, while gregarious females showed wider heads. With all of the mean values lower in nymphs, only femur length showed significant difference between the adults and nymphs (Figure 1D–F). The sex ratio of the sample was not biased (χ^2^_2_ = 2.847, *p*-value = 0.241).

**Figure 2:**
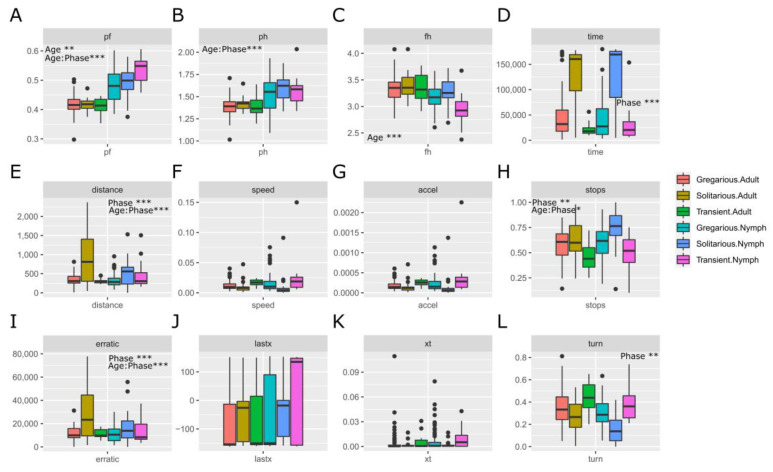
Comparison of the morphometric and behavioural traits used for building the models between solitarious, transient, and gregarious adults and nymphs of the desert locust *Schistocerca gregaria* used in this work. (**A**–**L**): pronotum-femur index, pronotum-head index, femur-head index, elapsed time, total distance, average speed, average acceleration, stop ratio, erratic movement, last coordinate, choice by time and turn ratio, respectively. The *X* axis represents the samples (solitarious, transient, or gregarious adults or nymphs) and the *Y* axis is the value of the trait analysed. Abbreviations of the traits are as explained in Table 1. *: *p* < 0.01, **: *p* < 0.001, ***: *p* < 0.0001.

None of the morphometric indices showed statistically significant differences between the current gregarious and solitarious samples (Figure 2A–C). Nevertheless, the pronotum to femur (P/F) and femur to head (F/H) indices showed differences between nymphs and adults. Both indices showed interactive effects of phase and age: solitarious nymphs showed higher values for these indices compared to gregarious nymphs, while adults showed no differences between phases but lower values compared to nymphs (*p*-value < 0.001 in both cases).

As expected, unlike gregarious locusts that rapidly went to the side of the arena where the gregarious stimulus was placed, solitarious locusts moved more slowly and randomly within the arena; thus, they showed higher values for the behavioural variables associated with less determination, hesitation, and lethargy (Figure 2D–L). They showed significantly longer elapsed time (Figure 2D)—i.e., it took them longer to reach the stimulus side of the arena, compared to the gregarious locusts, and some did not reach that side of the arena for the full 3 min of the test. They also showed significantly more turns (Figure 2L), meaning that they were less determined, hesitated, and moved more randomly compared to the gregarious locusts. They also travelled significantly longer distances (Figure 2E), stopped more (Figure 2H), and showed more erratic movement (Figure 2I) than the gregarious locusts—more signs of less determination in heading towards the stimulus—although the differences in these three last variables were age-dependent. In addition, elapsed time and turn ratio showed differences between gregarious and transient individuals (*p*-values 0.036 and 0.009, respectively). Stops showed differences only for transient individuals, and a statistically significant interaction between age and phase was also seen for that variable: while solitarious nymphs took more time static compared to gregarious nymphs, this tendency was inverted in adults. The individual and mean values of the analysed variables are given in Supplementary Tables S1 and S2.

**Figure 3:**
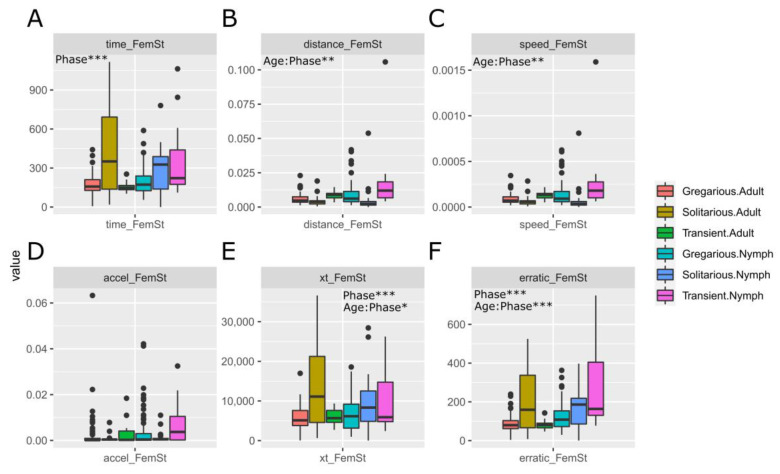
Effect of the normalization of the movement-related behavioural traits used for building the models (abbreviation of the variables are as specified in Table 1) by the femur length (FemSt) of the solitarious, transient, and gregarious adults and nymphs of the desert locust *Schistocerca gregaria* used in this work. (**A**–**F**): Elapsed time/femur length, total distance/femur length, average speed/femur length, average acceleration/femur length, choice by time/femur length and erratic movement/femur length, respectively. Significant effects and interactions, as determined by ANOVA, are shown with asterisks in each corresponding graph. *: *p* < 0.05, **: *p* < 0.01, ***: *p* < 0.001.

We checked whether the statistical differences would change after normalization by femur length of six behavioural variables that are related to movement and that could therefore be affected by locust size (i.e., elapsed time, total distance, average speed, average acceleration, C/T, and erratic movement) (Figure 3A–F). Average speed and acceleration continued to not show statistical differences between gregarious and solitarious locusts, and elapsed time and erratic movement continued showing differences between such locusts. However, distance no longer was associated with significant differences between gregarious and solitarious locusts, while C/T—which is the last coordinate reached divided by the time spent to reach it—showed differences between the gregarious and the transient locusts.

### 3.2. The Sg_extended_corrected Model Is Not Accurate, although It Does Distinguish between Solitarious and Gregarious Nymphs

**Figure 4:**
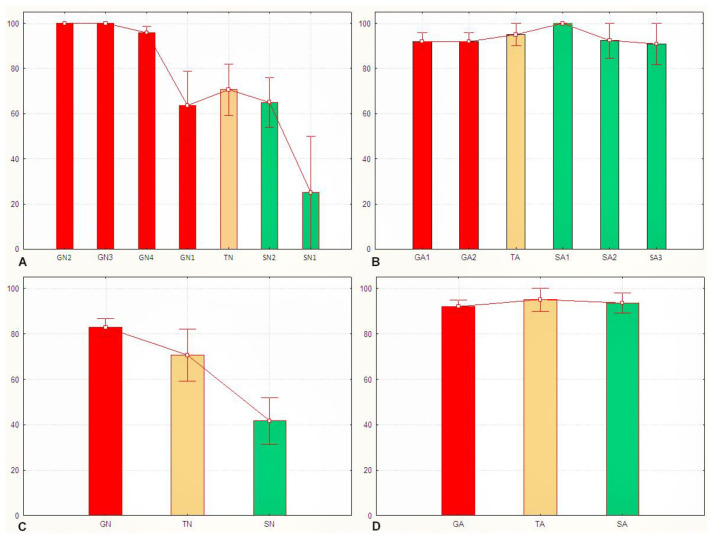
Gregariousness probability (P_greg_), shown as percentage in the *Y* axis, as obtained after application of the Sg_*extended_corrected* model from [27] to the solitarious (green), transient (orange), and gregarious (red) nymphs (**A**,**C**) and adults (**B**,**D**) of the desert locust *Schistocerca gregaria* used in this work. GA: gregarious adults, GN: gregarious nymphs, SA: solitarious adults, SN: solitarious nymphs, TA: transient adults, TN: transient nymphs.

Application of the *Sg*_*extended_corrected* model to *S. gregaria* nymphs allowed distinction between the solitarious and the gregarious ones. Accordingly, Figure 4A shows how solitarious nymphs had lower mean probability of being gregarious (P_greg_ mean ± SE = 0.65 ± 0.09) than gregarious nymphs (P_greg_ mean ± SE = 0.93 ± 0.03). Although the mean P_greg_ value for solitarious nymphs was higher than 0.5, the difference between solitarious and gregarious P_greg_ is statistically significant (Z-adjusted = 4.330, *p* < 0.0001). Indeed, the distribution of the P_greg_ values was clearly different between the solitarious and gregarious nymphs, although it had a relatively broad range, with some overlap observed (0.25 to 0.65 in solitarious nymphs, and 0.64 to 1.00 in gregarious nymphs). The standard deviations were clearly dependent on the sample sizes and, just as we found in [27], the model’s results are interpretable at the group and not the individual level (see Supplementary Tables S3–S5 for details).

For transient nymphs, the P_greg_ values were intermediate (0.71 ± 0.11), and statistics suggest that those nymphs became not significantly different from the solitarious group (Z-adjusted = −1.097, *p* = 0.273) and were no longer similar to the gregarious samples from where they came (Z-adjusted = 2.670, *p* = 0.008).

Taking all three independent nymph groups together (i.e., when increasing the sample size), the model shows even clearer separation of the gregarious, transient, and solitarious *S. gregaria* nymphs (Figure 4C).

Application of the *Sg_extended_corrected* model to adults gave mean P_greg_ values of 0.92 ± 0.06 for solitarious and 0.95 ± 0.05 and 0.92 ± 0.03 for transient and gregarious adults, respectively. Thus, as was the case in [27], it reproducibly failed to assign acceptable differences in P_greg_ values between solitarious and gregarious adult locusts: Z-adjusted = −0.271, *p* = 0.787 for solitarious versus gregarious adults, Z-adjusted = −0.213, *p* = 0.831 for solitarious versus transient adults, and Z-adjusted = −0.455, *p* = 0.639 for gregarious versus transient adults (Figure 4B,D and Supplementary Tables S4 and S5).

**Figure 5:**
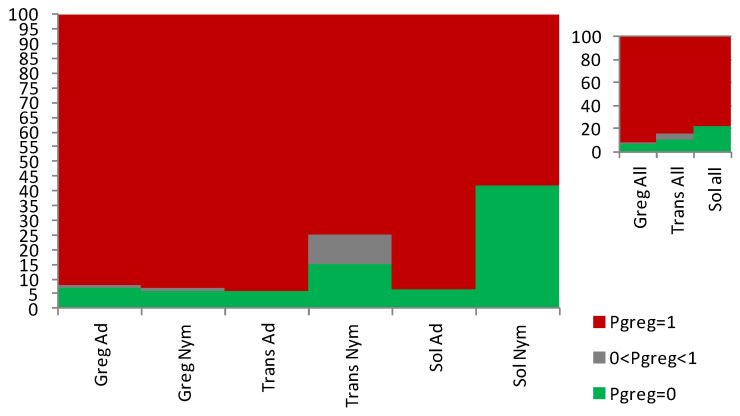
Proportions of individuals that show 0 (green), between 0 and 1 (grey), and 1 (red) as gregariousness probability (P_greg_), shown as percentage in the *Y* axis, as obtained after application of the Sg_*extended_corrected* model from [27] to the solitarious nymphs (Sol_Nym) and adults (Sol_Ad), transient nymphs (Trans_Nym) and adults (Trans_Ad), and gregarious nymphs (Greg_Nym) and adults (Greg_Ad) of the desert locust *Schistocerca gregaria* used in this work. Sol_All, Trans_all, and Greg_all are, respectively, the pooled solitarious, transient, and gregarious adult and nymph samples.

Overall, and although the adult samples sizes were larger than the nymph ones, when applied to both stages at once, the model discriminates between phases (Z-adjusted = 3.059, *p* = 0.002) but, as seen before, there is a significant difference between developmental stages (Z-adjusted = 2.378, *p* = 0.017). Based on the distribution of the P_greg_, Figure 5 confirms how this model works for nymphs but not for adults.

### 3.3. The Sg_non-morphometric Model Does Distinguish between Solitarious and Gregarious Locusts

**Figure 6:**
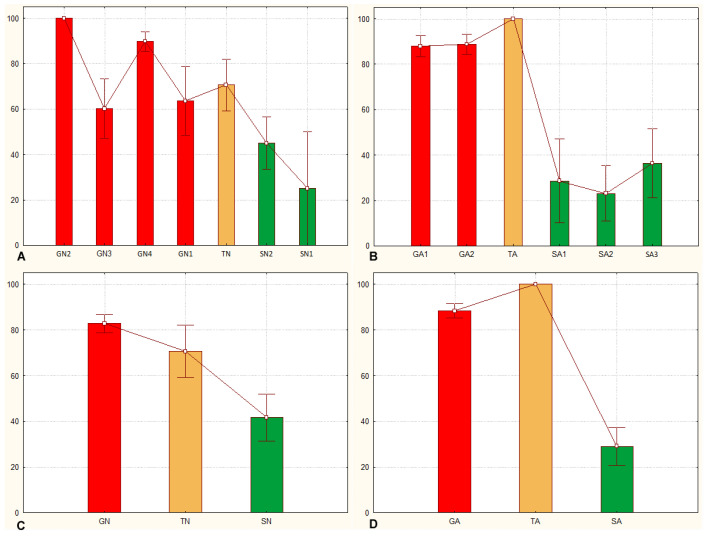
Gregariousness probability (P_greg_), expressed as percentage in the *Y* axis, as obtained after application of the *Sg_non-morphometric* model from [27] to the solitarious (green), transient (orange), and gregarious (red) nymphs (**A**,**C**) and adults (**B**,**D**) of the desert locust *Schistocerca gregaria* used in this work. GA: gregarious adults, GN: gregarious nymphs, SA: solitarious adults, SN: solitarious nymphs, TA: transient adults, TN: transient nymphs.

As in [27], the *Sg*_*non-morphometric* model did distinguish well between the solitarious and gregarious nymphs, with P_greg_ mean ± SE values of 0.45 ± 0.09 and 0.83 ± 0.04, respectively (Z-adjusted = 4.452, *p* < 0.00001). It also distinguished between the solitarious and gregarious adults, with P_greg_ mean ± SE values of 0.29 ± 0.10 and 0.88 ± 0.03, respectively (Z-adjusted = 6.825, *p* < 0.000001) (Figure 6A,B). As in the previous model, transient locusts showed intermediate and gregarious P_greg_ means for nymphs (0.88 ± 0.08) and adults (1.00 ± 0.00), respectively. However, this model suggests that the transient nymphs and adults remained not statistically different from the gregarious group from which they came and did not become similar to solitarious locusts (Z-adjusted = −2.134, *p* = 0.033 for solitarious versus transient nymphs, Z-adjusted = 1.160, *p* = 0.246 for gregarious versus transient nymphs, Z-adjusted = −4.829, *p* = 0.000001 for solitarious versus transient adults, and Z-adjusted = −1.623, *p* = 0.105 for gregarious versus transient adults).

The model therefore again distinguishes solitarious from gregarious locusts in both nymph and adult stages (Figure 6C,D and Supplementary Tables S4 and S5) and, as was the case when we used the *Sg_extended_corrected* model, the transient adults show high P_greg_ in the *Sg_non-morphometric* model.

**Figure 7:**
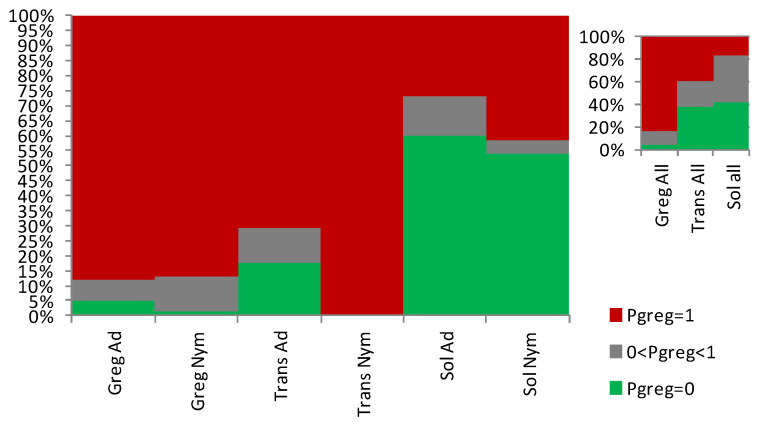
Proportions of individuals that show 0 (green), between 0 and 1 (grey), and 1 (red) as gregariousness probability (P_greg_), shown as percentage in the *Y* axis, as obtained after application of the *Sg_non-morphometric* model from [27] to the solitarious nymphs (Sol_Nym) and adults (Sol_Ad), transient nymphs (Trans_Nym) and adults (Trans_Ad), and gregarious nymphs (Greg_Nym) and adults (Greg_Ad) of the desert locust *Schistocerca gregaria* used in this work. Sol_All, Trans_all, and Greg_all are, respectively, the pooled solitarious, transient, and gregarious adult and nymph samples.

Overall, the *Sg_non-morphometric* model differentiates between phases better than the *Sg_extended_corrected* model (Z-adjusted = 8.062, *p* < 0.000001) while, contrary to the latter, it does not show any between-stage difference in performance (Z-adjusted = 0.889, *p* = 0.374). The distribution of P_greg_ further supports the better outcomes of the *Sg_non-morphometric* model both for nymphs and for adults (Figure 7).

## 4. Discussion

Locust outbreaks are a significant recurrent problem in several parts of the world, and outbreaks of the main pest locust, *S. gregaria*, are associated with the shift from the solitarious to the gregarious phase. The shift between phases occurs in response to changes in the environment (living conditions), and locusts of both phases show adaptations in the form of substantial differences in almost every aspect of their biology (see Section 1). In the absence of a clear qualitative or quantitative single morphological or behavioural marker that indicates the phase of a locust, some of the differences between solitarious and gregarious locusts could be combined so that we can infer the phase of the locusts or, at least, compare between locust groups or between different time points of the same locust group. This was thus far achieved using logistic regressions that combine different traits. The problem is that each single published work that differentiated between locust samples based on such methods used a different combination of traits and a different logistic regression formula (e.g., [16,25,26]). This not only implies spending time for building a new model for each experiment, it also means that the results might not be transferable or comparable between the different experiments and laboratories. Providing a time-saving tool is valuable for science, and standardising methods is a must. That is why we aimed in [27] at providing a solution to this problem in the form of an agnostic tool for time savings and for standardising the way solitarious and gregarious *S. gregaria* samples are categorised. However, the problem re-emerged after doubts were cast on the validity of such standardising tools [28], so that new models for research were required—thus again encountering the non-standardised realm—or the models that we suggested could be used under the risk of criticism on the basis that those models are doubtful or not valid.

Therefore, our aim in this study is to answer the question of the validity of those models, which if proven valid, would provide an even more trustworthy tool for solving the problem of the lack of standardised tools for *S. gregaria* sample categorization and phase assessment. That being said, we do not aim at characterising the phenomenon of locust outbreaks per se (an issue already approached in several earlier works [12,13,14,19,21,29,30,31,32,33,34,35,36,37,38,39] and that is still being analysed in current research). Neither do we aim at a simple repeat of a previous work; thus, we will not discuss every single result in the present work as, having successfully replicated our previous results, such discussion can be found in our earlier extensive work [27].

Nonetheless, we highlight that here, as in [27], preliminary analyses on the selected traits of adults and nymphs, males and females, and solitarious and gregarious *S. gregaria* showed expected size differences between males and females (with females generally larger) and between nymphs and adults (with adults generally larger). They also confirm that our solitarious and gregarious samples are indeed different (with both types of locust samples showing traits expected for their respective phase). They additionally reflect how no single morphological or behavioural trait is sufficient to distinguish between solitarious and gregarious *S. gregaria* groups.

The models that we test here introduce two novelties: normalization of the movement-related variables by the size of the locust and, in one model, inclusion of morphometric traits together with behavioural traits in the formula. Contrary to the insinuations in [28], we do not include colorimetry in the models, and we do not suggest that the models are applicable to other locust species (that is why we include “*Sg*” in the model names). In fact, we actually tested, proved, and suggested the contrary, and we suggested that similar studies should be carried out to determine the standardised features and models for assessing gregariousness in each species [27].

### 4.1. Should We Normalise by Locust Size?

This, in principle, should be as obvious as stating that dividing the distance travelled by the leg size of the traveller will allow more accurate comparison between the levels of activity of tall and short runners. Here, as in [27], we consider the fact that some movement (see behavioural) variables are a function of, and could be affected by, the animal’s size. Such an effect could distort the differences between solitarious and gregarious individuals. We corroborate this by showing how the variable distance (Figure 2E and Figure 3B), when not normalised by the animal’s size, shows a higher mean for solitarious locusts than for gregarious ones. Had we not normalised by femur length (a proxy of the animal’s size—as we explain in [27]), we would have had as a result and interpretation that the solitarious individuals walked greater distances in the observation arena, so they were more active than the gregarious individuals (which is contrary to what is proven and known about the differences between solitarious and gregarious *S. gregaria*). It is normalisation that attenuates the effect of the larger body size on the distance travelled by the solitarious locusts. Movement-related variables are undoubtedly a function of the leg size variable, so rather than introducing a leg-size effect (often vaguely described as morphology in [28]), the normalisation that we applied (a division) actually mathematically removes (or at least attenuates) the leg-size effect from the movement-related variables (often vaguely described as behaviour in [28])—introducing such an effect would be mathematically true had we added or multiplied by the leg size. So, for model building, we used the femur size (not a random morphological variable) for normalisation after explaining why it is the most suitable and valid proxy for leg and body size, and we normalised movement-related variables that we previously proved to be associated with *S. gregaria* phase (not a random behavioural variable). Given that the model’s regression coefficients are calculated based on the normalised movement-related variables, the weight (i.e., the regression coefficient) of each normalised variable is mathematically adequate for the values that that variable shows and for the association of such values with the locusts’ states. That being said, while larger individuals may well move longer distances, size is not the only reason solitarious individuals might travel larger distances than gregarious individuals. Solitarious locusts are more hesitant and lethargic (when they move, they wander about for a longer time, travelling greater distances in the observation arena). Gregarious locusts, however, quickly and directly move towards the stimulus (they take shorter paths and have a higher speed and acceleration). These characteristics reflect the direct movement seen in marching bands of gregarious locusts in the field.

### 4.2. Can Morphometry and Behaviour Be in the Same Model?

*S. gregaria*’s phase change involves either morphological and behavioural or only behavioural changes and is a dynamic phenomenon. Behaviour is the best indicator of phase state because locusts always change behaviour when they change phase. For its part, morphology can be an indicator of the phase, but this applies only to long-term solitarious and gregarious locusts. In addition, different *S. gregaria* samples will have morphological differences even when they are of the same phase, developmental stage, and sex.

At the same time, if one is to use variables in order to differentiate between two states, then one has to select the variables that significantly differentiate those two states and use as many variables as possible in order to be as accurate as possible. Hence, it is expected that the more variables that are associated with phase change that one considers, the closer one is to reality and to correctly inferring the phase of a locust sample. Using morphological and behavioural variables for distinguishing locust samples that differ in morphology and behaviour should therefore be a plus.

As an objection to introducing morphology, much was made of the potential situation of a locust that would be stimulated/induced into the gregarious phase and tested straightaway (non-long-term gregarious). It was claimed that because such locusts will not change morphology, using morphological variables in the model is erroneous [28]. That was quite surprising, given that in [27], we (i) explain that the models work at the group not individual level and, especially, (ii) we suggest using the *Sg_non-morphology* model, not the model that includes morphometry, for such locusts (i.e., those that do not change morphology).

### 4.3. Do the Models Do What We Want Them to Do?

Research on locust phase change is quite important and deserves standardised tools for one of its essential tasks—i.e., comparing locust groups. However, the message in [28] is that standardization of such tools is not possible. Moreover, the same author considered our models to be flawed and not recommended, and predicted that they “will not predict future observations as well as it appeared to predict on the present sample” [28]. These criticisms and predictions were based on theoretical evaluations (simulations) and some views and interpretations of proven and unproven concepts, but not on the actual application of the models to real locusts.

In experimental sciences, experiments are more trustworthy than interpretations of concepts, with the latter having to adapt to the empirical results and not the other way around—in fact, interpretation and indirect evaluation-based predictions themselves need testing.

Direct testing of the models by applying them to real locust samples is quick (a matter of minutes or, at most, a few hours), easy, and feasible (in [27], we provide the methods, formulae, and even a script to facilitate this task).

Here we re-evaluate and test the validity of the models as if we were working in a different laboratory. So, although the methods are exactly the same (as should be the case for a verification work), in the present study, the models are tested using additional independent samples of real locusts, and the researchers who carry out the observations and data collection are different from the ones in [27]; the researchers that apply the models are blind to the phase state of the analysed locusts.

In our original work [27], we suggested using the *Sg_extended_corrected* model (which includes morphometric variables) for comparing different *S. gregaria* nymph samples and the *Sg_non-morphometric* model (which does not include morphometric variables) for testing *S. gregaria* adults or the same *S. gregaria* nymphs at different time points (if they do not moult). This was successfully replicated in the present work, as (i) the *Sg_extended_corrected* model only predicts the phase for *S. gregaria* nymphs, and (ii) the *Sg_non-morphometric* model predicts the phase of both adults and nymphs of the same species.

Here, we successfully replicated the findings of [27], with the results that the two models are applicable only at the population (sample) and not the individual level and that distinguishing between samples of intermediate densities falls beyond these models’ sensitivity.

We therefore re-tested and corroborated again that the models built and initially tested in [27] can distinguish between solitarious and gregarious *S. gregaria* nymphs and adults. Although the models are useful for testing groups, but not single individuals, we highlight—as we did in [27]—that the experiments are conducted using samples (populations/groups) and not individual locusts, and that individual locusts might behave in a way that is not expected for their phase due to uncontrolled or even stochastic reasons. Thus, we confirm that the models suggested in [27] can be used for inferring the phase or for comparing samples/groups/populations of *S. gregaria* locusts (both different samples or the same sample between experimental times, e.g., when testing the effects of experimental manipulations such as the effect of drugs, gene silencing, etc., on the phase of *S. gregaria*). We thus reiterate that the use of these models would standardise and homogenise methodologies in benefit of reliable results and interpretations.

Testing, replication, and reproduction of the results are key to science, and science is based on hypotheses-driven results that are prone to testing and rejection and that should stand valid as long as they are capable of being reproduced. The models we suggest for inferring the phase of *S. gregaria*—for the sake of standardising methods between experiments and laboratories— were tested in two works, by different researchers, and using a total of 447 locusts pertaining to 25 different sets of *S. gregaria* locusts of different origin, densities, developmental stages, and sexes. The models consistently differentiate between solitarious and gregarious *S. gregaria* sets (see groups or populations). They, thus, should do so whenever they are used, a fact that allows us to confidently suggest their use for the benefit of standardising methodologies and saving time. The models were previously successful tested in [27], and when reapplied to our data from [27] by others, the models gave the same results, leading to the assertion that the models “appeared” to work “well” in our 2017 samples [28]. The successful testing of the models on the additional samples used in the current work hence further consolidates their validity and, contrary to the assumptions in [28], they are now even more valid than they were when initially suggested in 2017.

We highlight, as we did in [27], that the *Sg_non_morphometric* model works on adult and nymph samples and produces a better outcome compared to the *Sg_extended_corrected* model, which we suggest for different nymph samples and the same nymph sample if it changes morphometry (i.e., moults) between tests. Had we to choose only one model, we would recommend the *Sg_non_morphometric* model over the *Sg_extended_corrected* model. Of course, both models work only for *S. gregaria* and are better in larger samples sizes (as shown by the results when we pool all the samples of the same phase together).

So, contrary to the message in [28], we have shown here that standardisation of the models for categorising solitarious and gregarious locusts is possible and that the models that we suggested in [27] do work and are expected to work for future samples. We therefore prove here that there is a solution to the serious problem of the lack of standardised tools for categorising *S. gregaria* locusts and assessing their phase status, and that solution is the use of the models that we suggest.

That being said, the models, capable of correctly categorising solitarious and gregarious *S. gregaria* samples as thus far they have proven to be, are useful for different sample types, are not equally valid, are not optimal, and can no doubt be improved. We would certainly applaud anyone who can rebuild and improve them using larger sample sizes. As these models are species-specific, we also encourage colleagues working on other species to build and standardise tools for their work.

## Figures and Tables

**Table 1:** Variables, their coefficients (β_variable_) and intercepts (β_0_), and the correction factor (c) of the models for estimating *Schistocerca gregaria*’s probability of being gregarious. Each model’s equation is as follows: η = intercept + (β_P/F_ × P/F) + (β_P/H_ × P/H) + … + (β_EM_ × EM). The probability of being gregarious is calculated as P_greg_ = e^ηc^/(1 + e^ηc^), where e is Euler’s number (2.718) and c is a correction factor. I: *Sg_extended_corrected*. II: *Sg_non-morphometric*.

Correction Factor and Variables of the Model’s Logistic Regression Formulae		Constants and Coefficients of the Model:	
		I	II
C		1/104	1
Intercept (β0)		−2.83 × 10^7^	1.12 × 10^4^
Pronotum-femur index	Pronotum-femur index (PF = pronotum dorsal length divided by hind femur length)	5.11 × 10^7^	0
Pronotum-head index	Pronotum-head index (PH = pronotum dorsal length divided by head width)	−1.69 × 10^7^	0
Femur-head index	Femur-head index (FH = hind femur length divided by head width)	9.39 × 10^6^	0
Choice	CH = a binary variable describing the side of the arena where the experimental animal was positioned at the end of the recording, with 0 being the blank side and 1 being the stimulus side	1.03 × 10^6^	8.45 × 10^3^
Elapsed time	ET = total time of the recording, maximum 3 min if the animal does not reach one side of the observation arena earlier	−3.53	5.30 × 10^−3^
Total distance	TD = total distance travelled by the experimental animal during the recording time	−6.68 × 10^2^	−21.3
Average speed	AS = average of dividing the distance increments by the time increments at each time frame	7.91 × 10^8^	−3.41 × 10^5^
Average acceleration	AA = average of dividing the speed increments by the time increments at each time frame	−2.53 × 10^5^	−1.47 × 10^4^
Last coordinate	LC = value of the observation arena’s X-axis coordinate where the animal was positioned at the end of the recording, ranging between 150 at the blank side and +150 at the stimulus side	−3.68 × 10^3^	−18.3
Choice by time	CT or XT = last coordinate divided by the elapsed time	1.90 × 10^7^	−6.46 × 10^4^
Stop ratio	SR = number of 67-ms time frames with no distance increment divided by the total number of time frames of the recording	−9.04 × 10^5^	−7.53 × 10^3^
Turn ratio	TR = number of time frames when the animal turned divided by total number of time frames of the recording. A deviation from the path is considered as turn if the angle increment between two consecutive time frames exceeds 9°, i.e., 5% of a full, 180° turn	−5.33 × 10^10^	2.04 × 10^7^
Erratic movement	EM = the summed product of the turn angle and the distance per time frame	29.1	0.435

## Data Availability

All data generated or analysed during this study are included in this published article and its Supplementary Information files.

## Supplementary Materials

The following supporting information can be downloaded at: https://www.mdpi.com/article/10.3390/insects15020102/s1, Table S1: Values of the variable used in the models per individual locust. Table S2: Mean values of the of the variable used in the models per category of locust age and phase. Table S3: Outcomes of the models (P_greg_) per individual locust. Table S4: Mean values of the models’ outcome (P_greg_) per locust cage. Table S5: Mean values of the models’ outcome (P_greg_) per category of locust age and phase.
